# The College of British Nursing

**Published:** 1917-07-28

**Authors:** 


### THE MATRONS' AND SISTERS' DEPARTMENT.

### THE COLLEGE OF BRITISH NURSING.

### Steady Progress, Over 6,000 Members Registered, a United and Whole-hearted Council, With the Support and Interests of the British Nursing Profession Behind it. Its Few Irreconcilable Opponents "with Selfish Ends to Serve."

LAST week we gave a full report of the interesting speech made by the Hon. Arthur Stanley, C.B., M.P., at the second annual meeting of the College of Nursing. That speech is one which we hope will be reprinted in full in pamphlet form and widely circulated amongst the nurses and all who are interested in them, our hospitals and kindred institutions throughout the Empire—that is, everybody of intelligence. If proof be needed that the College has now behind it the interest of all classes of trained nurses throughout the Empire and at the Front, the important questions propounded by those present, and others sent direct to the Chairman from many quarters, which collectively represent the views of the democracy of the British nursing world, may be accepted as convincing testimony. (See p. 323, THE HOSPITAL, July 21, 1917.)

Now that our nurse democrats have commenced, through the College, to voice their problems, we may conclude with certainty that trained nurses in increasing numbers are awakening to the value of great representative institutions under democratic government, which the Hon. Arthur Stanley and the Council are steadily developing, for the comfort, education, organisation, and protection of British nursing everywhere. A few detractors with axes to grind, who support each other by fairy tales whilst denouncing the College and talking of democracy with a capital D, are untiringly printing word-pictures of what British nurses should aim at, whilst their main efforts, if the world may judge by the results, are directed to sowing disunion amongst British nurses for their own ends, as the Germans, as we write, are sowing rebellion amongst the soldiers in Russia. It is well, therefore, that our cousins in the United States of America, especially those associated with the medical and nursing professions, there, should clearly realise, as is now recognised in Great Britain, that, were it not for the few sowers of disunion, who, Mr. Stanley is afraid, are working "rather to serve their own selfish ends," and who, "while agreeing to negotiate, are openly and secretly doing their absolute best to damage the people with whom they pretend to be working," the profession of British nursing would be, and in fact is, united. An agreed Bill, providing for the State Registration of Nurses, therefore, may be sanctioned by the Houses of Parliament within a very short space of time.

It is the fashion of the small body of irreconcilables to blow themselves out and swell with importance when committing words to paper, but the claims advanced have been so overdone, that they cease longer to attract serious attention from the nursing world in these islands. It might be well if they were to study Æsop's fable of the Shepherd Boy and the Wolf. They persistently ignore, on a question like the State Registration of Nurses, how the profession of nursing has changed and developed in the last two decades, and the consequent growth of opinion among all classes, including the public, the medical profession, hospitals and training-schools, and an ever-increasing number of working nurses, too. This change is the result of no single person's or group of persons' propaganda, but a natural development, due to the ever-increasing number of and the demand for certificated trained nurses in this country.

Mr. Stanley made a strong point when he insisted, as he has done throughout, that the Council's intention and wish is to be made elective under the Bill for the Registration of Nurses, to the maximum extent. Indeed, so far as the College of Nursing is concerned, the aims and object are to get the full representation of British nurses in the government of the College by election, as soon as possible. In accordance with constitutional practice in this country, there will always have to be a certain number of nominated members, on any Council, set up by Parliament, who will be nominated probably by the Privy Council, the British Medical Association, and others; but the spirit and actual basis of government of the College of Nursing will be democratic and elective. It is essential to insist on these points, because the small party of irreconcilables, with ends of their own to serve, continuously circulate the falsehood, that the College scheme provides for the widespread control of the nursing profession by the laity—that is, their employers. British nurses, like their American cousins, have a deep attachment to their *Alma Mater*, their training-school, and no educated nurse, animated by the highest standards and self-respect, has any feeling but great appreciation, because she has every reason to respect those who taught her her business, and has reverence for the school which granted her the certificates, which qualify her to pursue with success the profession of nursing. Mr. Stanley convincingly and clearly testified, after eighteen months' experience, that the Council of the College have shown entire freedom from prejudice, an absence of personal aims, and that they are absolutely and whole-heartedly united in the task to build up the whole of its organisation, and to deal impartially and wisely with the many problems which have already presented themselves for solution. In speaking of the Council, Mr. Stanley properly insisted that the one endeavour of every member had been and was to do what is best for the nursing profession.

Unity being assured, the call for clear thinking on the part of every British nurse has arisen. Every trained nurse should get clearly into her head the meaning of the words "the College." In speaking of the amalgamation between the College of Nursing, Ltd., and the Royal British Nurses' Association, when the new by-laws are approved by the Privy Council, the words "the College" will mean the Royal British College of Nursing, that is, the amalgamated body. It appears that the first time "the College" was used in this sense was in a speech by Sir Henry Burdett printed in The Hospital of the 7th inst., p. 281, under the heading "The College of Nursing." No one can accurately use the words "the College" in speaking of the Privy Council's approval of the amalgamation, or afterwards, except to include both the College of Nursing, Ltd., and the Royal British Nurses' Association, with the Registers of both. The time has come for British Nurses to end controversy, discountenance gossip, and get on with the College and State Registration, whilst the way is open to immediate success. Delays are dangerous, and prompt and united action to-day means victory for British Nurses all along the line.

The mainspring and continued efforts of the College are to obtain adequate protection for the certificated, trained nurse. The first step to that end is the recognition by Parliament of the College with its Register, with State protection, which will safeguard and secure the position of the trained nurse. That will mean a fixed standard of training for all certificated nurses, which will not be lowered, but have a tendency to be raised, if anything, as time passes and knowledge increases. The demand for State Registration is now practically universal, and were it not for the few irreconcilables, Nurse Registration, Statutory Recognition, and Protection for the Certificated, Trained Nurse would be an accomplished fact before the year is out. The position of the College, as Mr. Stanley insisted, is clear. It is always ready and willing to enter into conference, and to discuss the terms of any Bill that its Council may wish to bring in, with any Society that can properly claim in any way to represent nurses. The one and only aim of the College and its supporters, is, to get a State Registration Bill, and to get it in the terms that will be best for the nurses themselves.

